# Assessment of dens invaginatus and its characteristics in maxillary anterior teeth using cone-beam computed tomography

**DOI:** 10.1038/s41598-021-99258-0

**Published:** 2021-10-05

**Authors:** Manal Alkadi, Rahaf Almohareb, Soad Mansour, Mohamed Mehanny, Raed Alsadhan

**Affiliations:** 1 Division of Endodontics, Department of Clinical Dental Sciences, College of Dentistry, Princess Nourah Bint Abdulrahman University, Riyadh, Saudi Arabia; 2 Department of Basic Dental Sciences, College of Dentistry, Princess Nourah Bint Abdulrahman University, Riyadh, Saudi Arabia; 3 Department of Oral Medicine and Diagnostic Sciences, College of Dentistry, King Saud University, Riyadh, Saudi Arabia

**Keywords:** Anatomy, Medical research

## Abstract

This cone-beam computed tomographic (CBCT) study aimed to evaluate the prevalence of dens invaginatus (DI) and its characteristics in maxillary anterior teeth in a Saudi population. A total of 505 CBCT scans were evaluated, including a total of 2790 maxillary anterior teeth. The patients’ demographic data, including age and sex, were recorded. The presence of DI and the related characteristics including bilateral occurrence; DI type according to Oehlers classification; and presence of periapical radiolucency, open apex, and/or nearby impacted teeth were analyzed. The associations between DI and the other factors were analyzed using the chi-square and fisher exact tests. DI was detected in 7.3% of the patients and 1.6% of the teeth examined. Most of the DI-affected teeth were maxillary lateral incisors (76.1%), followed by mesiodens (19.6%) and maxillary central incisors (4.3%), while no DI was observed in the maxillary canines. Bilateral DI was found in 24.3% of the affected patients. Oehlers type I DI was the most frequent (80%). Periapical radiolucencies, open apices, and nearby impacted teeth were observed in 10.9%, 4.3%, and 30% of the invaginated teeth, respectively. DI was significantly associated with tooth type (*P* < 0.0001) but not with sex (*P* > 0.05). Although most of the DI cases are limited to the crown, CBCT imaging is essential for DI evaluation and management.

## Introduction

Dens invaginatus (DI) is a developmental anomaly characterized by invagination of the enamel organ into the dental papilla before calcification of the dental tissues^1,2^. The prevalence of this anomaly has been reported to range from 0.3 to 10% of teeth and 0.25% to 26.1% of individuals examined^2^. The maxillary lateral incisors are the most frequently affected teeth. However, any tooth can be affected^1–5^. Several etiological factors have been described to explain the occurrence of DI, which have been primarily related to external forces on the tooth bud during tooth formation, such as adjacent tooth bud, trauma, infection, focal growth acceleration, and focal growth retardation of the tooth bud^6^. Additionally, genetic factors have been suggested^1^, and associations with some genetic disorders have been reported^5^.

Various classifications have been proposed to address the different presentations and severity of DI. Among these, the Oehlers classification was the most commonly used^7^. According to the Oehlers classification, type I DI is localized within the crown and does not extend beyond the cementoenamel junction (CEJ). In type II, the DI extends apically beyond the CEJ; however, remains as a blind sac within the root. In type III, the invagination penetrates through the root and eventually opens as an independent apical or lateral foramen. In type II, the DI may or may not communicate with the pulp, whereas in type III, there is usually no pulpal communication.

DI generally lacks any clear observable clinical signs. Therefore, it is not readily detected during routine clinical examinations and can remain undiagnosed in many situations. In most cases, DI is detected accidentally on radiographs obtained for other purposes^5^. As DI creates a potential path for irritants and microorganisms to the pulp, it is believed to increase the risk of pulpal and periodontal diseases, internal resorption, and adjacent teeth impaction^5,6^. The treatment of DI depends on the severity of invagination and the presence or absence of pulpal and/or periapical diseases. DI treatment options include preventive filling; pulpotomy; root canal treatment including conventional treatment, apexification, or pulp regeneration; apical surgery; and extraction^8^.

Advancements in imaging modalities used in endodontics, such as the use of three-dimensional (3D) cone-beam computed tomography (CBCT), have not only improved the diagnostic ability of teeth affected by DI, but also aided greatly in treatment planning and management of these cases^9^. Unlike conventional two-dimensional (2D) intraoral radiographs, CBCT allows visualization of the 3D morphology of DI, and thus provides more details about this anomaly, including its exact site of emergence and extension, course of penetration into the tooth, and whether it is associated with apical pathosis^5,10^. Previous studies have mainly assessed DI using 2D periapical and/or panoramic radiographs^1–4,6,11–13^. To date, limited studies have utilized CBCT to investigate the prevalence and characteristics of DI^5,14,15^. Therefore, this study aimed to investigate the prevalence of DI in maxillary anterior teeth in a Saudi population using CBCT. Additionally, DI characteristics were assessed, including DI type, site of occurrence, bilateral occurrence, and whether it was associated with apical pathosis and/or nearby impacted teeth.

## Materials and methods

CBCT scans of 505 patients obtained from the archives of the Department of Oral Medicine and Diagnostic Sciences, College of Dentistry, King Saud University, Riyadh, Saudi Arabia, from January 2017 to June 2019 were retrospectively reviewed. CBCT scans were obtained for different purposes, according to established guidelines for CBCT use (the AAE/AAOMR joint position statement), and none was obtained for the purpose of this study. All CBCT scans were acquired using the Planmeca ProMax 3D device (Planmeca, Helsinki, Finland) with the following acquisition parameters: 0.2-mm voxel size, 90 kV, 11 mA, and 15 s. Scans with both small and large fields of view were included. In addition, clinical records of the patients were reviewed; and demographic, orodental, and medical data were collected.

### Ethical approval and informed consent

After research registration, the present retrospective study was exempted from review by the institutional review board (IRB) at Princess Nourah Bint Abdulrahman University, Riyadh, Saudi Arabia, with reference number 20-0282. All the included patients provided written informed consent before obtaining the CBCT scans as part of their management. The consent included their approval to use and publish their data for educational and scientific research purposes.

### Sample size estimation

The sample size was calculated according to the formula suggested for the sample size estimation in prevalence studies^16^. With a 95% confidence level, a 3% margin of error, and maximum expected individual prevalence of 10%^17^; the minimum required sample size was estimated to be 385 individuals.

### Image assessment

Two experienced examiners, an endodontist and an oral radiologist, independently evaluated the CBCT images. Inter-examiner reliability was tested by evaluating the CBCT scans of 50 maxillary incisors independently by each examiner. After 1 month, the same teeth were evaluated to determine intra-examiner reliability. Any disagreement between the two examiners was discussed to reach a consensus. The images were viewed using the Planmeca Romexis Viewer software (Planmeca, Helsinki, Finland) on an HP Z420 workstation (HP, Palo Alto, CA, USA) on a 30-inch color LCD monitor (Barco MDCC-6130, Beneluxpark, Kortrijk, Belgium) with a resolution of 3280 × 2048 pixels in a dimly lit room. The axis lines representing the three planes (coronal, sagittal, and axial) were realigned along the coronoapical, mesiodistal, and buccopalatal axes of each tooth before evaluation. The contrast and brightness of the images were modified to ensure optimal visualization.

### Inclusion and exclusion criteria

Since the maxillary anterior teeth accounted for more than 90% of the DI-affected teeth reported in the literature^1–5,14,15^, and full-mouth scans were not available in adequate numbers; patients with CBCT scans showing at least the entire maxillary anterior region were included in the study. Patients with CBCT images of poor quality or scans not showing the entire maxillary anterior region and those with incomplete records were excluded. Additionally, individual teeth with root fillings, post and/or crown, or any metallic material that obscured adequate visualization of the natural tooth morphology were excluded from the evaluation. Supernumerary teeth were included in this study. However, for any scan to be included, a minimum of three normal maxillary anterior teeth, including a minimum of one lateral incisor, had to be present and fulfill the inclusion criteria.

### DI detection and assessment of its characteristics

The existence of DI was determined when any of the following was observed:

1. Evidence of radiopacity extending over the cingulum pit, beyond the radiopaque outline of the enamel, into the dentin
2. Well-defined radiolucency surrounded–completely or partially–by a radiopaque outline, extending beyond the outline of the enamel into the dentin.

When the presence of DI was determined, the following characteristics were evaluated and recorded:

1. Tooth type
2. DI type according to the Oehlers classification was as follows: type I, when the DI was limited to the crown and did not extend beyond the CEJ; type II, when the DI extended beyond the CEJ but did not perforate or communicate with the periodontium; and type III, when the DI extended beyond the CEJ and perforated through the root apically or laterally. To determine the level of CEJ (cutoff point between types I and II), a line connecting the buccal and palatal CEJ was drawn in the sagittal plane. If the DI did not extend beyond that line, it was considered type I. Otherwise, it was considered type II or III according to the aforementioned criteria. Sagittal sections were selected as they were the most helpful and reliable for determining the maximum DI extension.
3. Unilateral/bilateral presence of DI
4. The presence/absence of periapical radiolucency (PARL) according to the CBCT periapical index developed previously^18^. PARL was considered to be present if it had a diameter of > 0.5 mm in any CBCT plane (score: 1–5).
5. The presence/absence of an open apex. This factor was only assessed in teeth that had passed the normal time for complete root development and apical closure. An apex was considered open if it had a mesiodistal diameter of > 1 mm, regardless of the etiology of the open apex, which might include arrested root development, pathological root resorption, or combination of these conditions.
6. The presence/absence of nearby impacted teeth.
7. The shape of the invagination and any observed effects on pulp and/or root canal morphology.

### Statistical analysis

Descriptive statistics (frequencies, percentages, and means) were used to describe the prevalence of DI and its characteristics. The chi-square test and fisher exact test with the Bonferroni correction for multiple comparisons were used to assess the associations between presence of DI or DI type with other factors such as sex and tooth type. The intra- and inter-examiner reliabilities were determined using the Cohen kappa test. The significance level was set at *P* < 0.05.

## Results

A total of 505 patients with 2790 maxillary anterior teeth were included in the study. Of these, 287 were females and 218 were males. The ages of the patients ranged from 8 to 70 years, with a mean age of 30 years.

The frequency and percentage of DI according to sex and tooth type are shown in Table 1. Dens invaginatus was detected in the CBCT scans of 37 patients (prevalence, 7.3%), of whom 19 were females and 18 males, with no significant difference between the two sexes (*P* = 0.48). The mean age of the affected patients was 28 years (range, 8–69 years). Of the 2790 evaluated teeth, DI was observed in 46 teeth (prevalence, 1.6%) with the maxillary lateral incisors constituting the highest proportion of the affected teeth (76.1%), followed by mesiodens (19.6%) and maxillary central incisors (4.3%). No DI was detected in the maxillary canines. The DI prevalence among different tooth types was significantly different (*P* < 0.0001). The DI prevalence in mesiodens was significantly higher when compared with that in any other tooth type (*P* < 0.0001). Additionally, lateral incisors had significantly higher prevalence of DI than central incisors or canines (*P* < 0.0001), while no significant difference was observed between central incisors and canines (*P* = 0.50).

**Table 1:** Frequencies and percentages of DI-affected patients/teeth according to sex and tooth type.

	Sample (N)	DI-affected patients/teeth (N)	DI-affected patients/teeth (%)
**Sex**			
Female	287	19	6.62
Male	218	18	8.26
Total	505	37	7.33
**Tooth type**			
Maxillary central	922	2	0.22
Maxillary lateral	918	35	3.81
Maxillary canines	907	0	0
Mesiodens	43	9	20.93
Total	2790	46	1.65

There were 43 mesiodens detected in the study sample. Among them, 9 teeth were DI-affected, including 2 teeth that were multi-invaginated, in which more than one DI were observed in the same tooth. The prevalence of DI in normal dentition (excluding supernumerary teeth) was 5.7% in patients and 1.3% in teeth.

**Figure 1:**
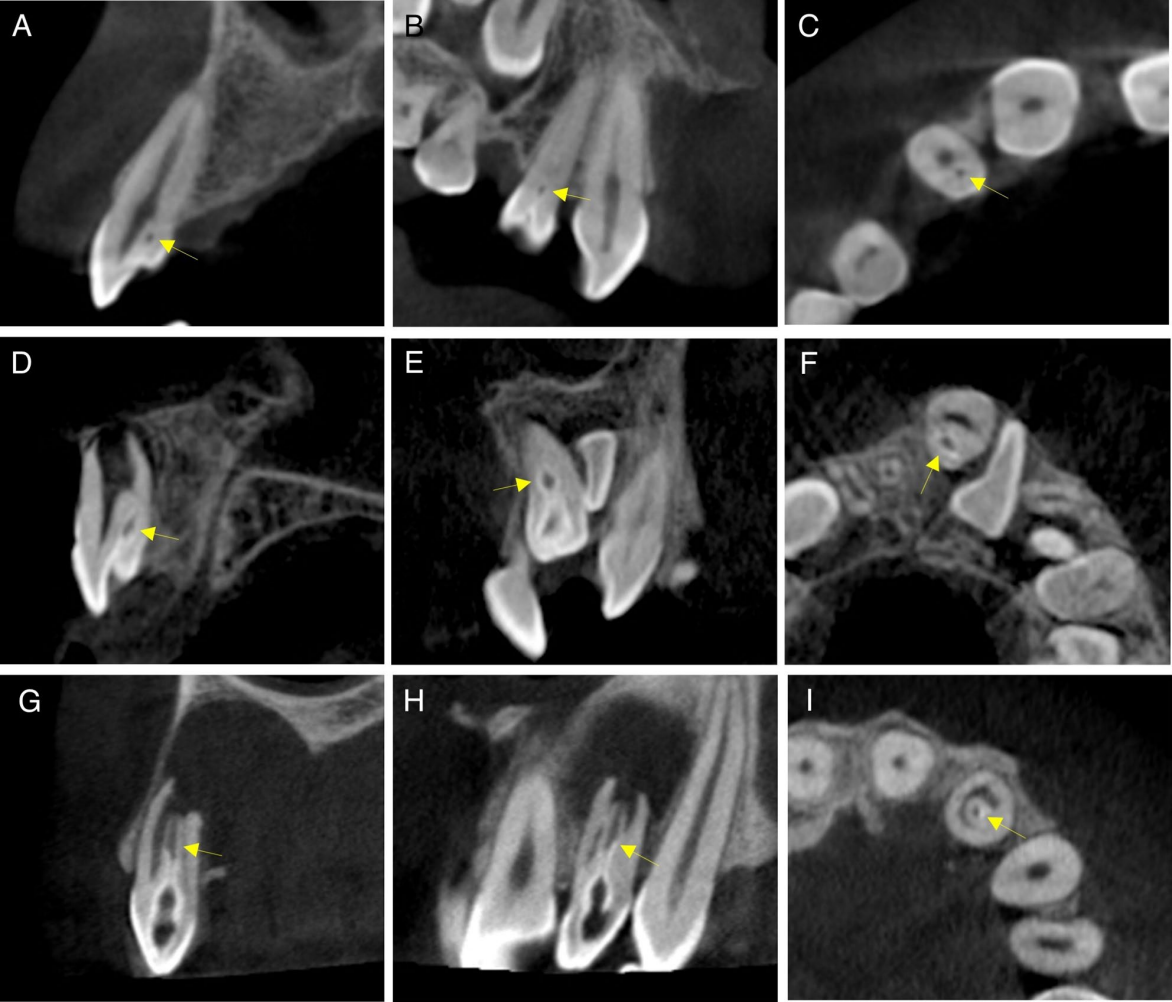
Representative CBCT images of the three DI types (yellow arrows). (**a**–**c**) Maxillary lateral incisor with DI type I. (**d**–**f**) Mesiodens with DI Type II. (**g**–**i**) Maxillary lateral incisor with DI type III. (**a**, **d**, **g**) Sagittal sections. (**b**, **e**, **h**) Coronal sections. (**c**, **f**, **i**) Axial sections.

The distribution of the DI cases according to sex, tooth type, bilateral occurrence, and Oehlers classification is presented in Table 2. Bilateral DI was detected in 24.3% of the affected patients. Most of the DI were Oehlers type I (80%), followed by type II (17.8%) and type III (2.2%). In one invaginated tooth, the crown was not completely developed, and the extent of invagination could not be determined. Thus, the DI type was considered as “not-applicable”, and the tooth was excluded from the DI type analysis. DI type was not associated with sex, nor tooth type (*P* > 0.05). Figure 1 shows representative images of each DI type.

**Table 2:** Distribution of DI-affected patients/teeth according to sex, tooth type, bilateral occurrence, and Oehlers classification.

	DI-affected patients/teeth (N)	% of the total DI-affected patients/teeth
**Sex**		
Female	19	51.4
Male	18	48.6
Total	37	100
**Tooth type**		
Maxillary central	2	4.3
Maxillary lateral	35	76.1
Maxillary canines	0	0
Mesiodens	9	19.6
Total	46	100
**Bilateral occurrence**		
Unilateral	28	75.7
Bilateral	9	24.3
Total	37	100
**Type of DI**		
Type I	36	80
Type II	8	17.8
Type III	1	2.2
NA	1	NA
Total	46	100

*NA* Not applicable.

Five of the 46 DI-affected teeth (10.9%) showed PARL. In one of them (with DI type I), deep caries was identified, and for other 2 teeth (with DIs type I and II, respectively), a history of dental trauma was reported. Additionally, among the 5 teeth that showed PARLs, 2 teeth were associated with open apices (4.3%) and had DIs type II and III, respectively. Based on the radiographic features observed, the open apex in one tooth appeared to be associated with arrested root development, while in the other one, it appeared to be caused by inflammatory apical root resorption. Table 3 shows the presence of PARL and/or open apex according to the DI type.

**Table 3:** Frequencies and percentages of PARLs and open apices according to DI type.

DI type	N	Teeth with PARL		Teeth with open apices	
		N	%	N	%
I	36	3	8.3	0	0
II	8	1	12.5	1	12.5
III	1	1	100	1	100
NA	1	0	0	NA	NA
Total	46	5	10.9	2	4.3

*NA* Not applicable.

Impacted teeth, whether single or multiple, were found near 14 DI-affected teeth (30%). Of the latter, 7 teeth (50%), including 6 mesiodens and 1 lateral incisor, were impacted themselves in addition to adjacent impacted teeth, and 9 teeth (64%) showed multiple nearby impacted teeth. The observed impacted teeth included central incisors (29%), canines (26.5%), mesiodens (26.5%), lateral incisors (12%) and premolars (6%).

In 30% of the invaginated teeth, a discontinuity was observed in the radiopaque outline of the DI. This was apparent in a single or multiple CBCT sections. The DI was teardrop in shape in 33 teeth (72%) and fissure-like in six teeth (13%), while only minor invagination beyond the cingulum pit was observed in seven teeth (15%). In parallel with the DI morphology and extension, the root canals were observed to bifurcate and branch around the DI in 36 teeth (78.3%) (Fig. 1A,D), appeared C-shaped in two teeth (4.3%) (Fig. 1I), and were normal in eight teeth (17.4%). CBCT sagittal sections were found to be the most useful for DI detection and evaluation.

The Cohen kappa test revealed high intra- and inter-examiner reliability with the values of 0.95 and 0.91, respectively.

## Discussion

As for other anatomical variations that may pose an impact on the pulpal and/or periapical health, the early identification and management of DI can be critical to improve the prognosis of the affected teeth. The present study assessed the occurrence and characteristics of this anomaly in maxillary anterior teeth in a Saudi population using CBCT. Although DI can occur in any tooth, the vast majority of the cases were encountered in the upper anterior teeth, and the reported prevalence in other tooth types was very low (0%–1%)^2–6,11,12,14,15^. Therefore, the present analysis focused on the assessment of DI in the maxillary anterior teeth.

DI was detected in 7.3% of the patients in the current study. This was slightly lower than the prevalence reported in a Turkish population (10.7%)^5^, in a CBCT-based study that also included supernumerary teeth. Considering the normal dentition, DI was observed in 5.7% of the patients. This was also lower than the prevalence reported by two CBCT studies in Chinese and Tunisian populations, with values of 8.5% and 12.5%, respectively^14,15^. Although these studies included posterior and mandibular teeth, the comparison can still be valid as the prevalence of DI in these teeth was quite low. Multiple studies have evaluated DI using periapical and/or panoramic radiographs, and the reported prevalence in individuals examined ranged from 0.17% to 12%^3,4,6,11,12^. In a sample of Saudi patients, DI was detected in 10% of the patients^17^. A higher value (37%) was recently reported in an Israeli population^13^. These variations may be related to different ethnic groups, methods of assessment, and/or diagnostic criteria.

In the present study, DI affected both sexes equally, which is consistent with the findings of previous studies^3,5,6,11,13,15^. However, other studies have found males to be more affected^4,14^.

The teeth most frequently affected by DI in this study were the maxillary lateral incisors, followed by the mesiodens and central incisors. Maxillary lateral incisors were consistently the most frequently affected teeth in all previous studies^1–6,11,12,14,15^. In the current analysis, mesiodens teeth constituted almost 20% of the teeth affected by DI. Although multiple case reports described DI in mesiodens teeth^19–24^, only two studies have been identified in which mesiodens were evaluated and accounted for 9% and 29% of the DI-affected teeth, respectively^5,25^. The latter was a non-prevalence study. The reason for this may be related to the relative lack of clinical implications of DI in mesiodens, as extraction is usually the eventual treatment. However, knowledge about the presence of DI in supernumerary teeth may provide additional insights and aid in better understanding the etiology of this anomaly. In the present study, central incisors accounted for 4.3% of the invaginated teeth, while none of the evaluated canines were affected. Similar results were observed in previous investigations^5,6,12,14,15^, in which maxillary central incisors were the second most frequently affected, followed by canines. In contrast, two studies reported the presence of DI in maxillary canines, but not in maxillary central incisors^3,4^. These differences could be attributed to the different populations studied.

In the current study, DI was observed bilaterally in 24% of the affected patients. No predominance was found in the literature for either the bilateral or unilateral occurrence of DI. Some studies reported higher percentages of bilateral DI^6,12,14,15,26^, while others found unilateral DI to be more frequent^3–5,11,25^. Yet, a recent study reported equal occurrence of the two patterns^13^. It has been suggested that bilateral DI can be associated with other dental anomalies, including gemination, microdontia, taurodontism and dentinogenesis imperfecta^6^. In contrast, the implication of local factors was proposed for the unilateral DI occurrence^13^. Since bilateral DI is not an unusual finding, it is recommended to examine the contralateral tooth in cases where DI is detected in a certain tooth.

Evaluating the depth and complexity of DI is essential for determining appropriate management. In this study, the Oehlers classification was used owing to its low complexity, wide acceptance, and clinical relevance. Based on the Oehlers classification, type I DI was the most frequently observed (80%), followed by type II (17.8%), and type III (2.2%). This pattern is consistent with that commonly observed in the literature^4–6,12–14^. However, one study found DI type II to be the most frequent, followed by type I and III^15^.

The narrow invagination associated with DI can enhance microbial retention^8^. Moreover, it has been indicated that the underlying enamel and dentin of the invagination might be thinner and structurally defective compared to those of the unaffected areas^1,13^. This may further enhance bacterial penetration and lead to pulpal and/or periapical diseases. In the present study, PARLs were observed in 10.9% of invaginated teeth; and specifically, in 8.3%, 12.5% and 100% of type I, II, and III DI-affected teeth, respectively. Similarly, previous studies found PARLs to be mainly associated with type III DI (33%–100%), and less frequently with type II (0%–10%) or type I (0–2.5%)^5,6,11,12,14,15^. Type III is the most severe and directly communicated with the periodontium, which leads to a higher chance of periapical involvement. However, it should be noted that the absence of observed PARL in the invaginated teeth does not exclude the possibility of pulpal disease. Additionally, many of the DI-affected teeth with periapical involvement might have been endodontically treated earlier, and thus were excluded from this study. After all, it should be considered that these findings are only descriptive and not implying a cause-effect relationship between DI and PARLs. Therefore, other etiological factors of PARLs such as deep caries independent of DI, dental trauma, cracks or periodontal disease are not excluded. In fact, of the five DI-affected teeth that showed PARLs in the study sample, deep caries was identified in one tooth, and a history of dental trauma was reported for other two teeth.

In some of the DI-affected teeth, the radiopaque outline of the DI was missing in some regions. This may be owing to the variable structure of the enamel in the DI, which may be thinner and hypomineralized compared to the normal one, as reported previously^1^.

Impacted teeth were found near 30% of the invaginated teeth, a finding that has been observed previously^5,15^. This may be due to a common etiological factor for the two conditions and may support the etiological theory of external forces during tooth development^5,27^.

It was observed that the more severe the invaginations, the more effect they produced on the anatomy of the pulp chamber and/or the root canal. For instance, in DI type I with only minor invagination beyond the cingulum pit, a tendency of the pulp chamber to branch palatally beneath the DI was observed. This branching was aligned with the shape and direction of DI. In more severe invaginations, as in types II and III, this effect appeared more distinct as the pulp bifurcated around the DI and resulted in two pulp horns and/or root canals, or even a C-shaped canal. The association of DI with a C-shaped main canal has also been observed previously^14,28^.

To the best of our knowledge, this is one of the few studies that utilized CBCT to evaluate DI prevalence and characteristics, and the first in a Saudi population. Additional CBCT-based studies with larger samples and different populations are recommended.

The current study has some limitations. This was a retrospective radiographic study, in which no correlations with clinical or histological findings were assessed. Additionally, it was based on a convenience sample; thus, it may not be representative of the population. All the patients included in this analysis had presented with indications for CBCT imaging^29^, which included teeth with complex morphology and dental anomalies. Therefore, the reported prevalence of DI may have been overestimated. However, another factor that may reduce the reported prevalence compared with the actual prevalence should be considered. Owing to the higher susceptibility of the teeth with DI to caries and pulpal disease, it is anticipated that a considerable number of these teeth had been previously restored and/or endodontically treated, and thus excluded from this study.

## Conclusion

Based on the evaluated CBCT scans, the prevalence of DI was 7.3% in individuals and 1.6% in maxillary anterior teeth in a Saudi population. Of the normal maxillary anterior teeth, the lateral incisor was the most frequently affected. Most invaginations were confined within the crown, and the bilateral occurrence of this anomaly was not uncommon. CBCT imaging can be an indispensable tool for proper diagnosis and management of DI and should be considered when the presence of this anomaly is suspected.

## Supplementary Information

Supplementary Information.

## Data Availability

The data that support the findings of this study are available on request from the corresponding author.
